# A case series: cytomegalovirus retinitis following autologous hematopoietic cell transplantation: a call for early detection and aggressive management

**DOI:** 10.3389/fmed.2025.1693928

**Published:** 2025-10-17

**Authors:** Yanhan Zhou, Mengyun Liu, Juntao Zhang, Ying Lu, Peipei Ye

**Affiliations:** ^1^Department of Hematology, The Affiliated People’s Hospital of Ningbo University, Ningbo, Zhejiang, China; ^2^Institute of Hematology, Ningbo University, Ningbo, Zhejiang, China; ^3^Department of Ophthalmology, The Affiliated People’s Hospital of Ningbo University, Ningbo, Zhejiang, China; ^4^Ningbo Clinical Research Center for Ophthalmology, Ningbo, Zhejiang, China

**Keywords:** autologous hematopoietic cell transplantation, cytomegalovirus retinitis, immunosuppression, case series, ophthalmologic surveillance

## Abstract

**Background:**

Cytomegalovirus (CMV) retinitis represents a rare yet vision-threatening opportunistic infection following autologous hematopoietic cell transplantation (auto-HCT), typically arising in the setting of profound iatrogenic immunosuppression. While CMV reactivation is a well-documented post-transplant complication, the occurrence of retinitis remains uncommon, and its clinical course and risk profile in auto-HCT recipients are inadequately characterized. Our findings underscore a novel association with delayed immune reconstitution and propose a strategy for enhanced surveillance and preemptive intervention.

**Case presentation:**

Between November 2019 and January 2025, six patients who underwent auto-HCT for a range of hematologic malignancies were diagnosed with CMV retinitis. The median age at presentation was 63 years. All patients were seropositive for CMV-IgG prior to transplantation. The universal presenting symptom was visual impairment, manifesting as either decreased visual acuity or blurred vision. CMV DNAemia was detectable in four (66.7%) patients at diagnosis. Bilateral ocular involvement was noted in four (66.7%) cases. 66.67% patients had received high-dose corticosteroid therapy, and five (83.33%) had been treated with monoclonal antibodies prior to the onset of retinitis. A consistent immunological finding was persistent lymphopenia with markedly low CD4^+^ T-cell counts (median 167 cells/μL). Management primarily involved intravitreal ganciclovir injections in the majority of affected eyes. Three patients (Patients 1, 4, and 6) experienced improved visual outcomes, while the other three (Patients 2, 3, and 5) had worsened vision. The all-cause mortality rate was 83.3% (5/6 patients), with fatalities attributed to concurrent infections, hemorrhage, and underlying disease progression.

**Conclusion:**

CMV retinitis is an emerging and serious complication after auto-HCT, strongly correlated with prolonged immunosuppression–particularly from T-cell-depleting monoclonal antibodies and corticosteroids–resulting in sustained CD4^+^ T lymphopenia. To enable early detection and intervention that may improve visual outcomes, we advocate for proactive ophthalmologic surveillance in patients–particularly those with high-risk persistent CMV DNAemia, severe lymphopenia, or any ocular symptoms (irrespective of systemic CMV DNAemia status). This surveillance should comprise extended immune monitoring and routine fundoscopic examinations to facilitate timely diagnosis.

## Introduction

Autologous hematopoietic cell transplantation (auto-HCT) remains the standard of care for patients with newly diagnosed multiple myeloma, high-risk or relapsed lymphoma, and other hematologic malignancies (1). Post-auto-HCT complications, particularly infections, are significant clinical concerns (2). While bacterial and fungal infections are most frequently encountered post-transplantation, cytomegalovirus (CMV) reactivation still presents a serious threat to transplant success in patients with hematologic malignancies, despite limited emphasis in management guidelines (3). Following allogeneic hematopoietic stem cell transplantation (HSCT), CMV reactivation occurs in 30%–80% of patients and may manifest as multiorgan involvement, including hepatitis, pneumonia, gastroenteritis, and retinitis (4, 5). Among these complications, CMV retinitis is exceptionally rare, observed in only approximately 0.2%–5.6% of allogeneic hematopoietic stem cell transplantation (allo-HSCT) recipients (2, 6, 7). This complication is associated with a dismal prognosis, with mortality exceeding 50% and a high risk of irreversible blindness, necessitating prompt intervention (8, 9). Notably, CMV retinitis after auto-HCT is even more uncommon, with only two cases reported in the literature to date (8, 10). Concerningly, the increased use of monoclonal antibodies (mAbs) and glucocorticoids may have been correlated with delayed immune reconstitution post-transplantation, during which a rising incidence of CMV retinitis has been observed (11, 12). This temporal association suggests that these agents are emerging risk factors for this complication. Given the severity of CMV retinitis, its suspected link to contemporary therapies, and the notable absence of dedicated prognostic studies in this patient population, this report describes the clinical characteristics and management outcomes of six patients with hematologic malignancy who developed CMV retinitis following auto-HCT, aiming to facilitate earlier recognition and improved outcomes for this devastating complication.

## Case report

**FIGURE 1:**
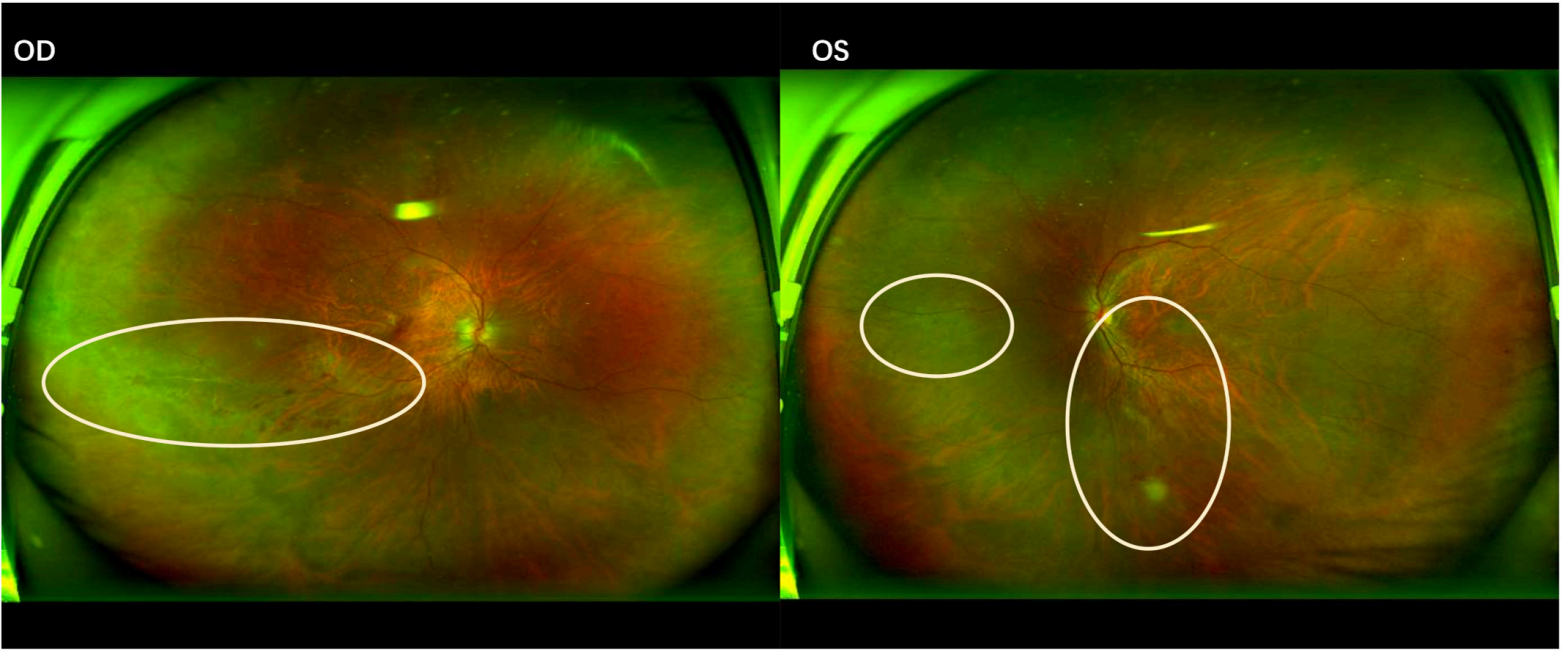
Fundus photograph of cytomegalovirus retinitis demonstrating a “hemorrhagic” appearance with lesions spreading centrifugally along the vascular arcades. OD (Right eye): Yellow-white necrotic lesions with associated retinal hemorrhages are present inferior to the optic disc. OS (Left eye): Yellow-white necrotic lesions and retinal hemorrhages are observed superior to, inferior to, and temporal to the optic disc.

**FIGURE 2:**
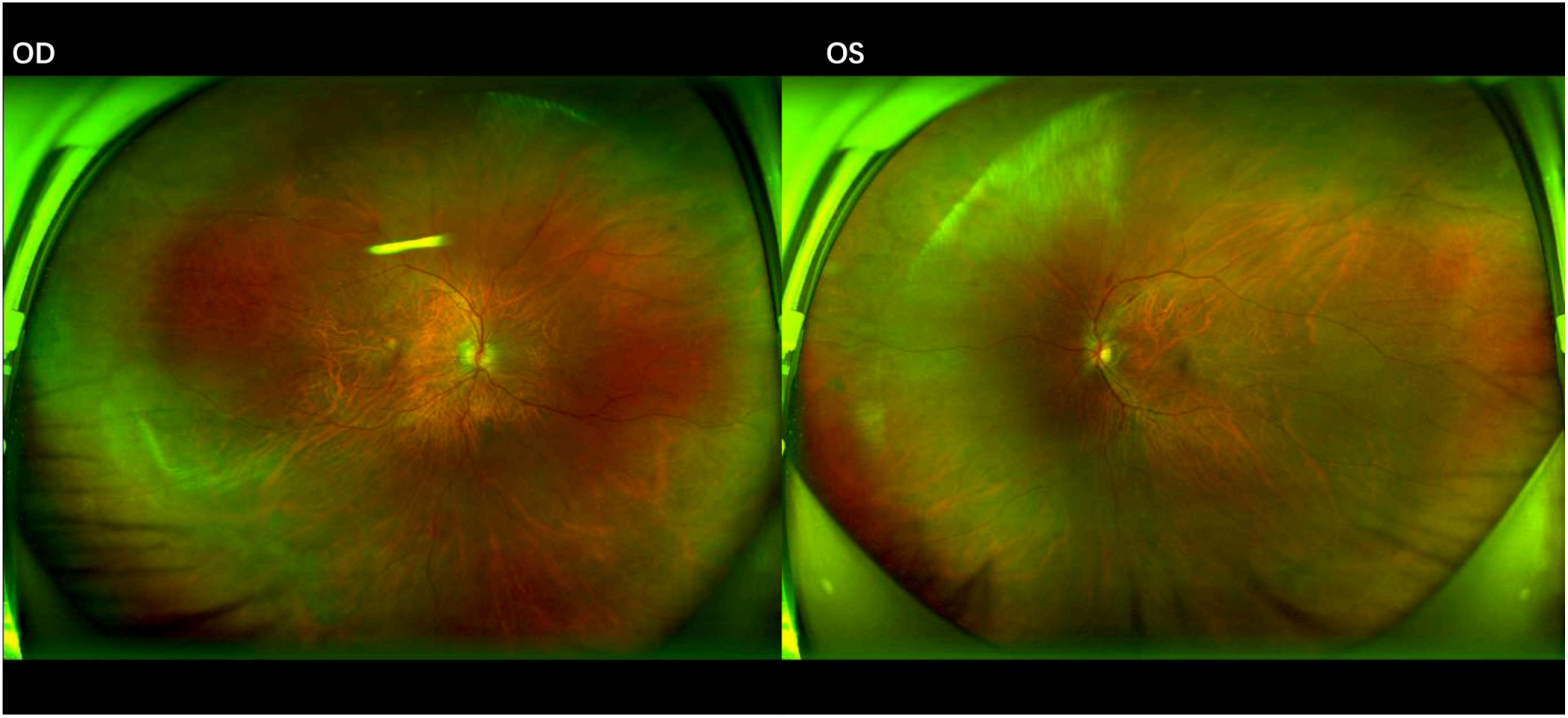
Fundus photographs after 1 month of anti-cytomegalovirus therapy. The lesions show signs of regression in both eyes, characterized by a reduction in the area of retinal necrosis and hemorrhage. The borders of the lesions have become well-demarcated with early signs of pigmentary changes, indicating the transition to inactive, scarred tissue.

**FIGURE 3:**
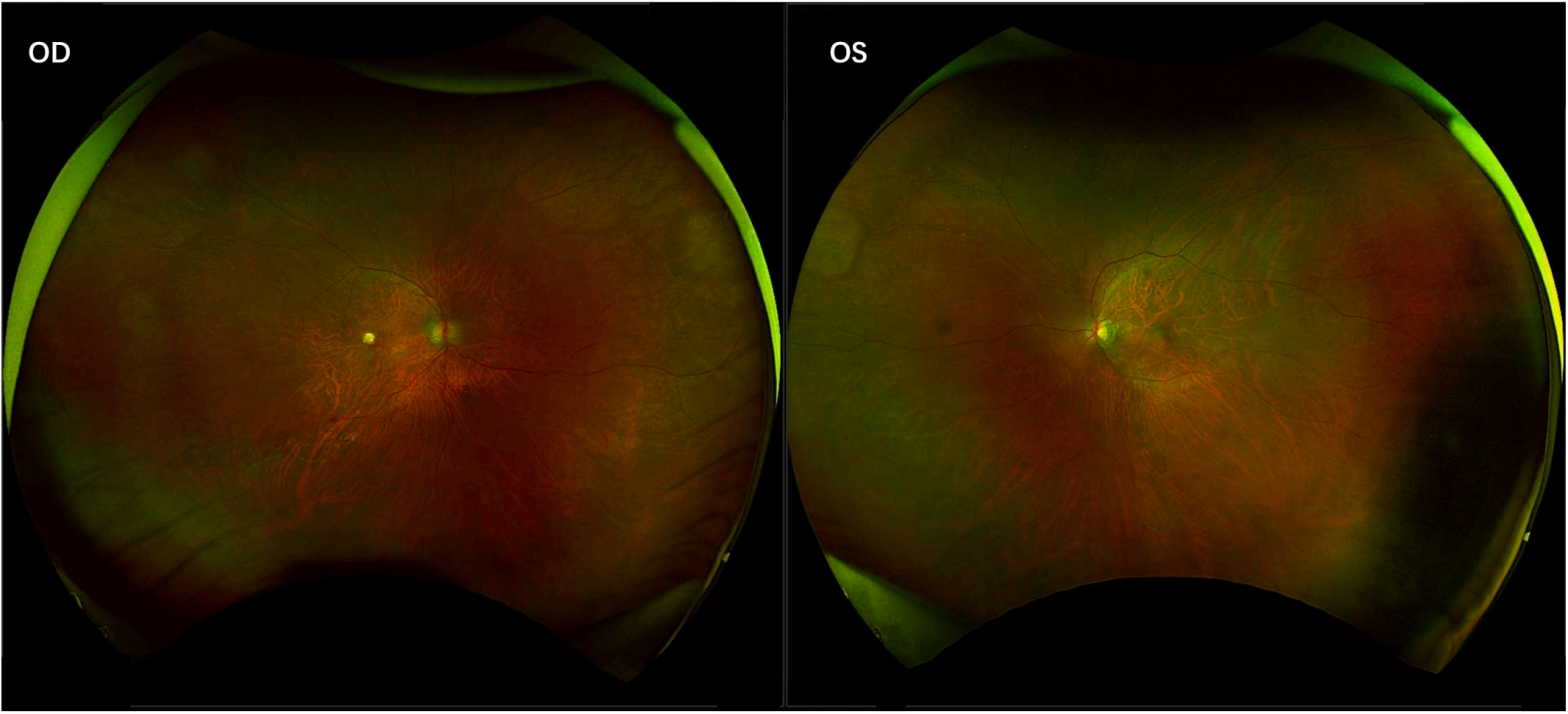
Inactive cytomegalovirus retinitis with chorioretinal scarring. The fundus photographs show the final, quiescent stage of the disease, characterized by well-demarcated areas of retinal atrophy and pigmentary changes.

Among a cohort of 295 patients who underwent auto-HCT between November 2019 and January 2025, six cases of CMV retinitis were identified. The diagnosis was established in all cases by characteristic ophthalmoscopic features and, where available, confirmed by detection of CMV DNA in aqueous humor via polymerase chain reaction. The underlying malignancies in these patients included angioimmunoblastic T-cell lymphoma, multiple myeloma, peripheral T-cell lymphoma, diffuse large B-cell Lymphoma and Mantle Cell Lymphoma. The median age of these patients was 63 years (range, 51–66 years). All patients were negative for CMV-IgM and CMV-DNA, while CMV-IgG positivity was present prior to transplantation. The median interval from transplantation to the diagnosis of CMV retinitis was 151 days (range, 64–720 days). At presentation, all patients reported visual symptoms, including decreased vision (*n* = 3) and blurred vision (*n* = 3). Four patients had concomitant CMV viremia or DNAemia, with a median peripheral blood CMV DNA level of 2.75 × 10^3^/mL (range, 2.12–5.64 × 10^3^/mL). Of these four, two developed invasive CMV disease involving other organs (pneumonitis). Additionally, one patient experienced a concurrent herpetic infection (herpes zoster). Conversely, the other two patients (of the total six) presented without detectable CMV viremia or DNAemia. Bilateral ocular involvement was observed in 66.67% of patients, with 10 eyes affected. At initial assessment, four of the 10 affected eyes had a best-corrected visual acuity (BCVA) of >0.1, another four eyes had a BCVA of ≤0.1, and data were unavailable for the remaining two eyes. Three patients experienced further complications, including rhegmatogenous retinal detachment and immune recovery uveitis. Table 1 summarizes the patients’ clinical characteristics, with representative fundoscopic findings from Patient 6 shown in Figures 1–3. For anti-CMV therapy, first-line treatment consists of intravitreal injections of ganciclovir at a dose of 2–4 mg. An induction regimen typically involves weekly injections administered over a period of 2–3 weeks. Following induction, the necessity for maintenance therapy is determined according to the activity of retinal lesions, as evaluated through serial funduscopic examinations. In cases where positive CMV viremia is detected, active intervention with intravenous antiviral agents is required for a minimum duration of 2 weeks. All affected eyes received this therapy, with a median of five injections per eye (range, 1–18 injections), reflecting this individualized treatment strategy. Two patients received intravenous ganciclovir for viremia (median duration: 19 days; range: 14–23 days). Of the remaining two viremic patients, one was unable to receive conventional intravenous antiviral agents–such as ganciclovir, foscarnet, or cidofovir–due to serious thrombocytopenia (blood platelet <20 × 10^9^/L) and renal insufficiency (creatinine >200 μmol/L). Furthermore, financial constraints precluded the use of intravenous immunoglobulin for immunomodulation. The other was lost to follow-up after transfer to another institution, resulting in missing treatment data. Over a median follow-up of 13 months (range, 2–52), five eyes (Patients #2, #3, and #5) suffered profound vision loss (deterioration in two eyes and no light perception in three). Notably, despite a short interval to presentation (10 days), Patient #5 still had a poor visual outcome. In contrast, accumulated experience enabled prompt intervention that prevented vision loss in the other five eyes (Patients #1, #4, and #6). Notably, five of the six patients (83.3%) died within 2–52 months of CMV retinitis diagnosis, from causes, including septic shock (*n* = 1), intracranial hemorrhage (*n* = 2), and progressive underlying hematologic malignancy (*n* = 2).

**TABLE 1:** Clinical characteristics and outcomes of six patients with cytomegalovirus retinitis after transplantation.

Patient number	Sex	Age, years	Systemic diagno-sis	Pre-trans-plant CMV sero-status	Type of mono-clonal antibody therapy before Tx	Time from last mono-clonal anti-body therapy to CMV retinitis Dx, days	Time from Tx to CMV retinitis Dx,days	Time from Symp-tom Onset to Oph-thalmic Evalua-tion, days	Eyes involved	Initial BCVA of affected eye	CMV organ disease	Ocular complica-tions	CMV viral copy number in the blood, ×10^3^/ml	CMV DNA Load in aqueous Humor, copies/mL	Intra-vitreal injec-tions	Visual out-come	Patient Out-come
1	M	66	AITL	IgG+	CD30	97	150	21	OD	0.1	None	RD	2.18	NA	14	Improved	death
2	F	64	MM	IgG+	CD38	322	720	365	OU	0.01/0.02	Pneumonia	OU IRU	3.39	NA	4	Worsened	death
3	F	66	PTCL	IgG+	No	No	91	63	OU	NA	None	OU IRU	2.12	NA	18	Worsened	death
4	M	62	MM	IgG+	CD38	395	332	31	OU	0.15/0.02	Pneumonia	None	5.64	NA	1	Improved	death
5	F	52	DLBCL	IgG+	CD20	165	64	10	OD	0.02	None	None	negative	NA	1	Worsened	death
6	M	51	MCL	IgG+	CD20	195	152	7	OU	0.8/0.8	None	None	negative	10.20	6	Improved	alive

M, male; F, female; AITL, angioimmunoblastic T-cell lymphoma; MM, multiple myeloma; PTCL, peripheral T-cell lymphoma; DLBCL, diffuse large B-cell Lymphoma; MCL, mantle cell lymphoma; CMV, cytomegalovirus; Tx, transplantation; Dx, diagnosis; BCVA, best-corrected visual acuity (decimal notation); OD, oculus dexter; OU, oculus uterque; NA, not available; RD, retinal detachment; IRU, immune recovery Uveitis; DNA, deoxyribonucleic acid.

Current guidelines indicate that extensive use of mAbs and high-dose corticosteroids (>20 mg prednisolone per day, ≥14 d of continuous or cumulative exposure) may contribute to the development of CMV retinitis (3). In this cohort, 83.33% patients received pre-transplant mAb therapy targeting CD30, CD38, or CD20. The median interval from the last dose of monoclonal antibody therapy to the diagnosis of CMV retinitis was 195 days (range,97–395 days). Notably, 66.67% patients received high-dose corticosteroids, with cumulative exposure calculated as the total prednisone-equivalent dose (mg). Such immunosuppression impairs lymphocyte proliferation and function, reduces the CD4^+^/CD8^+^ ratio, and delays immune reconstitution. Laboratory characteristics at diagnosis are summarized in Table 2. Prior to the diagnosis of CMV retinitis, lymphocytopenia (<1.50 × 10^9^/L) persisted for a median of 73 days (range, 23–332 days). At the time of diagnosis, the median absolute lymphocyte count was 1.06 × 10^9^/L (range, 0.63–2.83 × 10^9^/L). Mild lymphopenia (≤0.8 × 10^9^/L) was present in 50% (*n* = 3) of patients. The median CD4^+^ T-cell count was 167 cells/μL (range, 12–268 cells/μL), with 83.3% (*n* = 5) exhibiting counts < 200 cells/μL. Only one patient (16.7%) had CD8^+^ T-cells < 150 cells/μL, while all six patients demonstrated a CD4^+^/CD8^+^ ratio < 1.0, indicating significant immune imbalance. The median time from transplantation to diagnosis with CD4^+^ counts < 200 cells/μL was 126 days (range, 45–562 days), comparable to the median time from transplantation to diagnosis of CMV retinitis (151 days). The median gammaglobulin level was 5.52 g/L, with hypogammaglobulinemia (<5 g/L) present in two of six patients (33.33%). The median serum lactate dehydrogenase (LDH) level was 303 IU/L.

**TABLE 2:** Laboratory features at the time of cytomegalovirus retinitis diagnosis.

Patient number	Post-transplant lymphocyte count decline	Time from Tx to Dx of lymphocyte count reduction,days	Duration of CD4^+^ <200 cells/μL post-transplant, days	CD4^+^ T-cell count, cells/μL	CD8^+^ T-cell count, cells/μL	CD4^+^/CD8^+^ ratio	Lymphocyte counts,×10^9^/L	Gammaglobulin level,g/L	Lactate dehydrogenase, IU/L	Cumulative pred dose post-transplant, mg
1	Yes	140	150	170	244	0.69	0.73	6.64	349	195
2	Yes	332	562	268	562	0.48	0.80	3.07	274	150
3	Yes	102	102	86	1304	0.07	1.52	14.51	264	1102
4	Yes	44	308	12	47	0.25	0.63	5.11	293	1633
5	Yes	23	66	174	1110	0.16	1.31	5.94	313	570
6	Yes	39	45	163	614	0.26	2.83	4.46	378	724

Tx, transplantation; Dx, diagnosis; Pred, prednisone.

## Discussion

In a real-world cohort of 295 autologous hematopoietic cell transplantation (auto-HCT) recipients over a 5-year period, the overall incidence of CMV retinitis was 2.03%. This rarity contributes to its frequent under-recognition and subsequent preventable vision loss. It is noteworthy, however, that the risk is not uniformly distributed across all patients (13). A substantially higher incidence is observed in those exposed to mAb or high-dose corticosteroids under contemporary treatment protocols, particularly among individuals with persistent CD4^+^ lymphopenia–a marker of impaired immune reconstitution that warrants prolonged monitoring (14, 15). Furthermore, accumulated evidence underscores that prompt ophthalmologic referral upon the onset of ocular symptoms is critical for early diagnosis and visual preservation (13). In this study, profound and persistent CD4^+^ T-cell suppression (median, 167 cells/μL) accompanied by an inverted CD4^+^/CD8^+^ ratio may represent a key risk factor for CMVR retinitis. These findings are consistent with previous reports by Zhang et al. and Jeon et al., which associated severe T-cell dysfunction–marked by sustained depletion of both CD4^+^ and CD8^+^ T-cell subsets–with an increased incidence of CMVR following allogeneic hematopoietic stem cell transplantation (16, 17). Furthermore, this notion of immune compromise driving CMV retinitis pathogenesis is reinforced by Zhang J et al., who reported a positive correlation between the severity of immunosuppression and the degree of ocular inflammation (18, 19). However, direct mechanistic evidence linking combined CD4^+^ and CD8^+^ T-cell impairment to the development of retinal disease remains scarce. Moreover, no universally accepted threshold for CD4^+^ or CD8^+^ T-cell counts predictive of CMV retinitis has been established. To date, experimental proof is still lacking to demonstrate that concomitant CD4^+^/CD8^+^ lymphopenia represents a common pathogenic pathway in CMV retinitis. Nonetheless, monitoring these parameters remains clinically valuable for assessing individual immune reconstitution status and may help guide risk-stratified surveillance strategies. Moreover, 83.33% patients in this cohort had a history of monoclonal antibody therapy. As emphasized by Maschmeyer et al., antibody-based agents can induce depletion of B and/or T lymphocytes (20, 21). This immunosuppression results not merely from pharmacokinetic drug persistence, but from a protracted process of immune reconstitution requiring progenitor cell repopulation and functional recovery. This immunosuppressed state can persist for 12–23 months beyond therapy, thereby establishing a prolonged vulnerability window for opportunistic infections (22–24). In our cohort, the median 195 days (about 7 months) interval to CMV retinitis diagnosis following monoclonal antibody exposure corroborates this period of sustained immunosuppression. Moreover, when combined with high-dose corticosteroids, this leads to profound cellular immunodeficiency and markedly increases the risk of CMV reactivation (20, 25). These observations align with the 2024 NCCN guidelines, which underscore that concomitant use of targeted antibodies and corticosteroids significantly elevates the incidence of CMV end-organ disease (3, 26). Therefore, in patients undergoing such immunosuppressive therapies, a declining CD4^+^ T-cell count can serve as an early warning signal for CMV retinitis, indicating the urgent need for preemptive ocular screening and initiation of appropriate treatment (18, 27). Notably, two patients with CMV retinitis in our study presented without detectable CMV viremia or DNAemia. This echoes the work of Zhang et al., that CMV seronegative does not exclude ocular CMV infection, and that systemic viral load does not significantly correlate with CMV-DNA levels in the aqueous humor (18, 27). Consequently, systemic virological markers may not accurately reflect the extent of intraocular viral replication, highlighting the critical need for direct aqueous humor CMV-DNA quantification in the diagnosis of CMV retinitis (27, 28). Our study’s lack of complete aqueous humor assessment suggests that the true intraocular viral burden might be underestimated (27, 29). Consequently, aqueous humor CMV-PCR should be integrated into routine diagnostic workflows for CMV retinitis; this is essential to enhance detection sensitivity and facilitate targeted treatment (27).

Based on our findings and the existing literature, we propose a risk-stratified surveillance strategy for CMV retinitis after auto-HCT. We recommend active monitoring of lymphocyte subsets (CD4^+^, CD8^+^, and CD4^+^/CD8^+^ ratio) for at least 3–6 months following transplantation (8, 16–18). In addition, during periods of lymphopenia, elevated LDH levels should be monitored as a useful ancillary biomarker, as they correlate with immune dysregulation and an increased risk of opportunistic infection (30). Synthesizing evidence from the existing literature with data from our cohort, we propose the following key predictive laboratory thresholds: CD4^+^ count < 200 cells/μL, a CD4^+^/CD8^+^ ratio < 1.0, an absolute lymphocyte count < 1.0 × 10^9^/L (where ≤ 0.8 × 10^9^/L denotes heightened risk), and elevated lactate dehydrogenase (>300 IU/L) (8, 16–18, 30). The presence of these markers should trigger intensified surveillance. Recipients identified as high-risk–specifically those exposed to T-cell-depleting monoclonal antibodies or prolonged high-dose corticosteroids, and those with persistent lymphopenia (particularly CD4^+^ count < 200 cells/μL)–require extended and proactive ophthalmologic surveillance for 9–12 months. This should include a baseline dilated funduscopic examination within the first 3 months post-transplantation, followed by regular examinations (e.g., every 3–6 months) regardless of systemic CMV DNAemia status. Although the detection of CMV DNAemia mandates immediate ophthalmologic referral, its absence does not preclude the development of retinitis, as demonstrated in two of our patients. Therefore, vigilance must be maintained in high-risk patients even in the absence of viremia (27, 31). For patients who develop CMV DNAemia, intensive quarterly ophthalmologic follow-up should be instituted and continued for at least 9–12 months, even after clearance of viremia, to monitor for potential late-onset disease.

This study has several limitations. First, its retrospective design and small sample size, although notable for a rare complication, limit the generalizability of our findings and hindered our ability to perform a robust multivariate analysis of risk factors. Second, the absence of systematic aqueous humor CMV-DNA quantitative data–due to the loss of early records and the confirmation of diagnosis in some patients at external hospitals where only qualitative rather than quantitative values were provided, with just one of the six patients having specific numerical data–precluded a detailed analysis of the relationship between intraocular viral load and clinical outcomes. Third, as all patients were from a single center, potential selection bias cannot be excluded. Future multi-center prospective studies with standardized diagnostic and monitoring protocols are needed to validate our proposed surveillance strategy and better elucidate the immunologic mechanisms underlying CMV retinitis after auto-HCT.

## Conclusion

Cytomegalovirus retinitis is a rare but vision-threatening complication following autologous hematopoietic cell transplantation, often occurring in the context of profound and prolonged immunosuppression. Its development may be strongly associated with the use of monoclonal antibodies and high-dose corticosteroids, which delay immune reconstitution and lead to persistent CD4^+^ lymphopenia and immune dysregulation. The high mortality and irreversible visual impairment associated with this condition underscore the necessity of early detection and aggressive management. Therefore, regular and systematic monitoring of lymphocyte subsets, immediate ophthalmologic evaluation in response to CMV DNAemia or any visual symptoms–even in the absence of detectable viremia–, and regular long-term follow-up are essential to improving visual and overall outcomes in high-risk patients.

## Data Availability

The original contributions presented in the study are included in the article/supplementary material, further inquiries can be directed to the corresponding authors.
